# Unusual Case of Hepatic Amebiasis Without Recent Endemic Exposure: A Diagnostic Puzzle

**DOI:** 10.7759/cureus.94107

**Published:** 2025-10-08

**Authors:** Meghan E Acheson, Rachel E Garrity, Jada Bussey-Jones

**Affiliations:** 1 Department of Medicine, Grady Memorial Hospital/Emory University, Atlanta, USA

**Keywords:** abscess, amebiasis, amebiasis infection, entamoeba, entamoeba histolytica infection, hepatic abscess, hepatic lesions, protozoa and helminths

## Abstract

*Entamoeba histolytica* is a parasite endemic to many countries. While many people who are infected remain asymptomatic, serious complications can also occur. We report the case of a 25-year-old male immigrant from Guatemala who presented to the ED with epigastric pain and non-bloody diarrhea. Abdominal imaging revealed at least 10 hypodense liver lesions, prompting a further workup. He was subsequently diagnosed with invasive amebiasis infection due to *E. histolytica* and was initiated on appropriate antimicrobial therapy. This case underscores the importance of maintaining a broad infectious differential when evaluating hepatic lesions, even in patients without a recent travel history. This case is unique due to the presence of multiple amoebic abscesses in a patient living in a non-endemic region with a remote immigration history and potential sexual transmission. This report highlights a rare presentation of a relatively common infection in a patient without recent travel or known exposure, emphasizing the need for clinical vigilance in non-endemic settings.

## Introduction

*Entamoeba histolytica* is a protozoan endemic to many countries in tropical and subtropical regions, where it is a common cause of intestinal amebiasis. It is estimated that over 500 million people are infected worldwide, with most infections occurring in these endemic regions [1]. While rates of infection are estimated to approach 40% in parts of Central America and Asia, approximately 4% of the population in the United States is also estimated to be infected [2,3]. Infection with *E. histolytica* is primarily transmitted through the fecal-oral route; however, it can also be transmitted sexually, particularly through anal sex [4]. Mature cysts are excreted in the stool of an infected person and, once ingested by a new host, can form motile trophozoites that migrate through the body, causing invasive amebiasis [2]. Approximately 10% of the affected individuals develop symptomatic invasive amebiasis, and this invasive infection is responsible for over 100,000 deaths per year worldwide [5]. Clinical symptoms of invasive amebiasis infection often include bloody diarrhea, abdominal pain, and weight loss [2]. 

## Case presentation

Here we present the case of a 25-year-old male with no known past medical history and no previous interaction with the healthcare system who immigrated to the United States from Guatemala seven years ago. He presented to the emergency room with two days of epigastric abdominal pain and non-bloody diarrhea. The patient reported initial mild epigastric pain that progressed to severe pain with nausea and anorexia. 

The patient was born and raised on a ranch in Guatemala, where he had close contact with various farm animals such as ducks, chickens, pigs, cows, sheep, cats, and dogs. He also consumed meat, eggs, and unpasteurized milk from these animals. Since leaving Guatemala seven years ago, the patient had not traveled outside of Atlanta. He lived with two family members who were also from Guatemala, neither of whom had traveled outside of Atlanta recently. He worked in construction and endorsed occasional social alcohol use. He reported a single male sexual partner four months ago, who also emigrated from Guatemala five years before and had not traveled outside of the United States since. 

**Figure 1:**
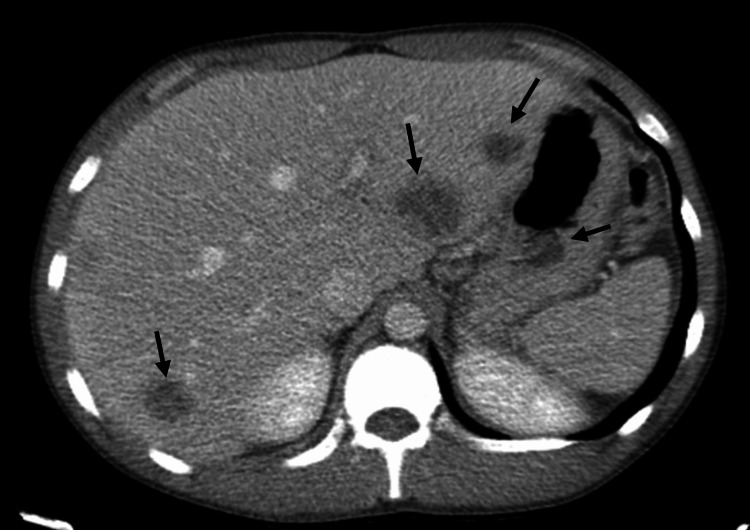
Multiple hypodense liver lesions on CT with contrast The patient’s initial CT on presentation to the ED revealed at least 10 bilobar hypodensities throughout the liver, raising concern for multifocal abscesses.

On arrival to the ED, the patient was febrile to 38°C, with a WBC count of 19.8 K/mcL and a neutrophil predominance of 84.2% without eosinophilia. Aspartate aminotransferase, alanine aminotransferase, and alkaline phosphatase were all within normal limits. His abdomen was soft, non-distended, and diffusely tender to palpation, worst in the left upper quadrant and epigastrium, without guarding, rebound tenderness, or rigidity. No hepatosplenomegaly was appreciated on physical examination. Contrast-enhanced CT scan of the abdomen and pelvis revealed at least 10 hypodense lesions with subtle rim enhancement scattered throughout the hepatic parenchyma, the largest of which measured 3.2 x 3.0 cm (Figure 1). Given concern for bacterial or parasitic liver abscesses, further infectious disease workup was initiated. 

**Figure 2:**
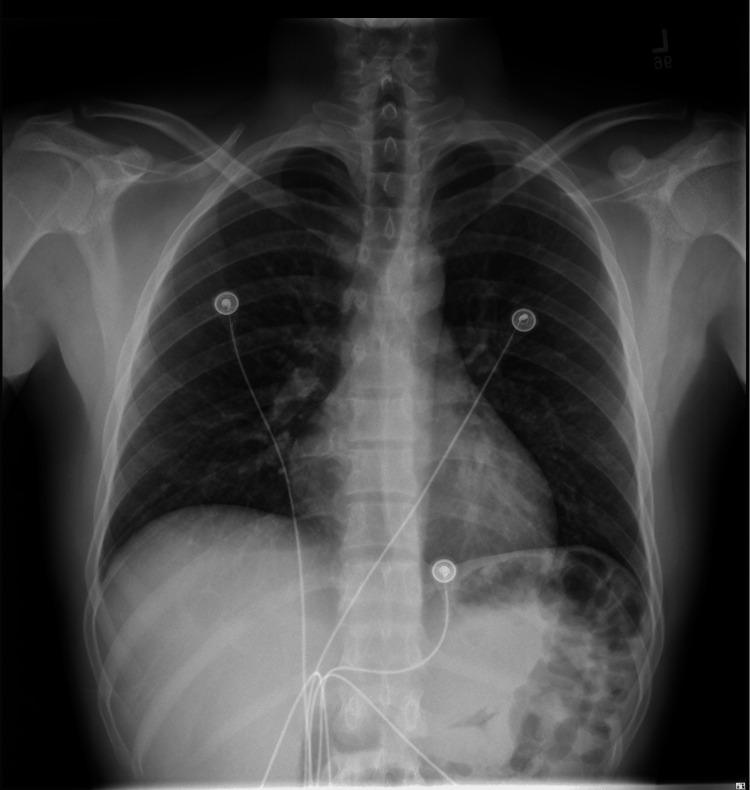
Chest X-ray The patient's chest X-ray revealed no acute abnormality.

Workup included a Biofire Gastrointestinal Panel, stool ova and parasites, *Echinococcus* antibody, *Cryptococcus* antigen, *Bartonella* antibody, *Brucella* antibody, and urine *Histoplasma* antigen. HIV, serologies for hepatitis A/B/C, and rapid plasma reagin were also obtained. Chest X-ray (Figure 2) was ordered to evaluate for tuberculosis (TB), given that TB is endemic in Guatemala. Hepatocellular carcinoma was also considered, as it can mimic liver abscesses; however, this was thought to be unlikely in a young patient with no past medical history and no evidence of cirrhosis. Empirical antibiotic therapy was started with ceftriaxone IV 2 g q24h and metronidazole PO 500 mg BID while awaiting diagnostic studies.

The results of the BioFire Gastrointestinal Panel came back the next day and were positive for both *E. histolytica* and *Campylobacter jejuni*. All other components of the infectious disease workup were negative. Ceftriaxone was discontinued, and his metronidazole dose was increased to 750 mg PO TID for 10 days. He was started on azithromycin 500 mg PO daily for three days for the *Campylobacter enteritis*. By hospital day four, the patient was afebrile with a WBC count of 12.9 K/mcL (Table 1) and was discharged in a stable condition. At a follow-up appointment in the infectious disease clinic two weeks later, he reported improvement in his abdominal pain and resolution of diarrhea. He was prescribed three days of nitazoxanide 500 mg PO BID for luminal cyst eradication. Nitazoxanide was selected instead of paromomycin due to formulary restrictions.

**Table 1:** WBC values during hospitalization

Hospital day	WBC count (reference range: 3.8-10.7 K/mcL)
Day 0	19.8 K/mcL
Day 1	18.3 K/mcL
Day 2	18.3 K/mcL
Day 4	12.9 K/mcL

## Discussion

This is a case of a 25-year-old male originally from Guatemala who was found to have amebic liver abscesses and was ultimately diagnosed with *E. histolytica*. This case is interesting, as the source of this patient’s infection is unclear. Although it is possible the patient contracted the parasite while living in Guatemala, where it is endemic, he had not traveled outside of Atlanta in over five years, and over 80% of patients with amebic liver abscesses develop symptoms within two to four weeks of exposure [6]. As this patient was previously sexually active with a man who was also from Guatemala, he reported no sexual activity in the three months preceding his diagnosis and that his sexual partner had not traveled outside of the United States in over five years. While much less common, cases of invasive amebic liver abscesses have previously been reported over 20 years since travel to an endemic area [7]. It is possible that this patient previously contracted the parasite in Guatemala and had a much longer incubation period than usual, or that he contracted the parasite from sexual contact with an asymptomatic infected partner in the prior months while living in the United States.

In addition, this case is unique due to the presence of multiple liver abscesses. While approximately 65%-75% of patients present with a solitary liver abscess, this patient had at least 10 hypodense liver lesions observed on CT [8]. While eosinophilia is often an important laboratory marker for parasitic infections, it is essential to note that infection with *E. histolytica* is not typically associated with eosinophilia, and none was observed in this case [9]. Therefore, when developing a differential, parasitic infections should still be considered even in the absence of eosinophilia to avoid missing a diagnosis such as *E. histolytica*. 

This case presented a diagnostic challenge, as the lack of recent exposure history, absence of recent travel to an endemic area, and presence of multiple lesions presented a confusing clinical picture. A comprehensive workup was ordered, including bacterial, fungal, and parasitic causes, which ultimately led to the diagnosis of *E. histolytica*. The inclusion of a broad differential enabled a timely diagnosis, and the patient experienced a significant clinical improvement after the initiation of empiric bacterial and parasitic coverage while studies were pending. 

Untreated invasive amebiasis is almost always fatal and is responsible for many of the over 100,000 annual deaths worldwide. With treatment, the mortality rate of invasive amebiasis is only 1%-3% [10]. The goal of treatment of *E. histolytica* amebiasis is to eradicate both the mature trophozoites that are actively causing amebiasis and the dormant cysts that have the potential to transform into infectious trophozoites. This patient was started on metronidazole 750 mg PO TID for a total duration of 10 days in addition to three days of nitazoxanide 500 mg PO BID for luminal cyst eradication. Traditional first-line therapy for amebic liver abscesses caused by *E. histolytica* is metronidazole 500-750 mg TID for seven to 10 days to eradicate the mature trophozoites, followed by a luminal agent such as paromomycin 500 mg TID for seven days. A luminal agent should be prescribed after the completion of metronidazole, as dormant cysts may persist in the intestines of 40%-60% of patients [6]. Paromomycin is typically the first-choice luminal agent used due to its well-known efficacy in luminal cyst eradication; however, it was not available on formulary for this case. Instead, the alternative nitazoxanide was prescribed, which has demonstrated up to 94% effectiveness at eliminating remaining *E. histolytica* organisms in stool samples [11]. 

## Conclusions

Although rare in the United States, infection with *E. histolytica* should remain on the differential for patients presenting with hepatic abscesses and suggestive epidemiological history. Without treatment, amebiasis can lead to serious complications and potentially death. Atypical presentations such as this case can occur years after exposure, with symptoms, including diarrhea and multiple hepatic abscesses. In addition to exposure history in endemic countries, it is also important to consider sexual history when evaluating potential sources, as this parasite can be transmitted sexually from asymptomatic carriers, even in non-endemic regions. 

## References

[1] Roro GB, Eriso F, Al-Hazimi AM, Kuddus M, Singh SC, Upadhye V, Hajare ST (2022). Prevalence and associated risk factors of Entamoeba histolytica infection among school children from three primary schools in Arsi Town, West Zone, Ethiopia. J Parasit Dis.

[2] Guillén N (2023). Pathogenicity and virulence of Entamoeba histolytica, the agent of amoebiasis. Virulence.

[3] Gunther J, Shafir S, Bristow B, Sorvillo F (2011). Short report: amebiasis-related mortality among United States residents, 1990-2007. Am J Trop Med Hyg.

[4] Billet AC, Salmon Rousseau A, Piroth L, Martins C (2019). An underestimated sexually transmitted infection: amoebiasis. BMJ Case Rep.

[5] Ximénez C, Cerritos R, Rojas L (2010). Human amebiasis: breaking the paradigm?. Int J Environ Res Public Health.

[6] Jackson-Akers JY, Prakash V, Oliver TI (2025). Amebic liver abscess. https://www.ncbi.nlm.nih.gov/books/NBK430832/.

[7] Nespola B, Betz V, Brunet J (2015). First case of amebic liver abscess 22 years after the first occurrence. Parasite.

[8] Pritt BS, Clark CG (2008). Amebiasis. Mayo Clin Proc.

[9] Kantor M, Abrantes A, Estevez A (2018). Entamoeba histolytica: updates in clinical manifestation, pathogenesis, and vaccine development. Can J Gastroenterol Hepatol.

[10] Stanley SL Jr (2003). Amoebiasis. Lancet.

[11] Rossignol JF, Kabil SM, El-Gohary Y, Younis AM (2007). Nitazoxanide in the treatment of amoebiasis. Trans R Soc Trop Med Hyg.

